# Interpopulational variation in the cold tolerance of a broadly distributed marine copepod

**DOI:** 10.1093/conphys/cou041

**Published:** 2014-10-07

**Authors:** Gemma T. Wallace, Tiffany L. Kim, Christopher J. Neufeld

**Affiliations:** 1 Friday Harbor Laboratories, University of Washington, Friday Harbor, WA 98250, USA; 2 Biology Department, Whitman College, Walla Walla, WA 99362, USA; 3 Department of Environmental Sciences, Northwestern University, Evanston, IL 60208, USA; 4 Quest University Canada, Squamish, BC, Canada VB8 0N8

**Keywords:** Chill-coma, cold tolerance, local adaptation, macrophysiology trend, *Tigriopus californicus*

## Abstract

Latitudinal trends in cold tolerance have been observed in many terrestrial ectotherms, but few studies have investigated interpopulational variation in the cold physiology of marine invertebrates. Here, the intertidal copepod *Tigriopus californicus* was used as a model system to study how local adaptation influences the cold tolerance of a broadly distributed marine crustacean. Among five populations spanning 18° in latitude, the following three metrics were used to compare cold tolerance: the temperature of chill-coma onset, the chill-coma recovery time and post-freezing recovery. In comparison to copepods from warmer southern latitudes, animals from northern populations exhibited lower chill-coma onset temperatures, shorter chill-coma recovery times and faster post-freezing recovery rates. Importantly, all three metrics showed a consistent latitudinal trend, suggesting that any single metric could be used equivalently in future studies investigating latitudinal variation in cold tolerance. Our results agree with previous studies showing that populations within a single species can display strong local adaptation to spatially varying climatic conditions. Thus, accounting for local adaptation in bioclimate models will be useful for understanding how broadly distributed species like *T. californicus* will respond to anthropogenic climate change.

## Abstract (precis)

Three metrics were used to study interpopulational differences in the cold tolerance of the intertidal copepod Tigriopus californicus. All three metrics showed a consistent latitudinal trend in which populations from colder northern latitudes had greater cold tolerance than those from warmer southern locations.

## Introduction

Temperature influences the performance and fitness of ectothermic animals, affecting their physiology, ecology, behaviour and evolution (Somero, 1997). Many species experience different thermal conditions throughout their habitat range, particularly those species that are distributed along broad latitudinal and altitudinal gradients. Organisms must maintain homeostasis in all environments, and while many organisms successfully tolerate a wide range of thermal conditions (Angilletta *et al.*, 2002), physiological trade-offs can arise between adaptations to high vs. low temperatures (Pörtner, 2002b; Angilletta *et al.*, 2003). Therefore, in species that have geographically heterogeneous thermal ranges, populations often evolve differences in thermal physiology that improve their fitness under local conditions (Huey and Kingsolver, 1989; Gaston *et al.*, 2009; Sanford and Kelly, 2011). Studying patterns of local adaptation in thermal biology can improve our understanding of how physiological traits evolve and differentiate at the population level (Chown *et al.*, 2004). In addition, local adaptation studies can provide insight into how species' distributions may change in response to anthropogenic climate change, because separate populations may have different ranges for thermal tolerance from the species as a whole (Kuo and Sanford, 2009; Hill *et al.*, 2011; Kelly *et al.*, 2011; Sanford and Kelly, 2011).

Numerous studies have investigated interpopulational differences in the thermal tolerance of terrestrial invertebrates (butterflies, Zeilstra and Fischer, 2005; *Drosophila*, Hoffmann *et al.*, 2001, 2002; David *et al.*, 2003; Sgrò *et al.*, 2010; Sisodia and Singh, 2010; isopods, Castañeda *et al.*, 2004, 2005; land snails, Gaitán-Espitia *et al.*, 2013; and mosquitoes, Zani *et al.*, 2005). Although less common, experimental laboratory approaches have also been successful in quantifying and qualifying local adaptation in marine organisms (Sanford and Kelly, 2011). Several papers have reported local thermal adaptation in marine invertebrates (copepods, Willett, 2010; Kelly *et al.*, 2011; gastropods, Hilbish, 1981; Kuo and Sanford, 2009; Zippay and Hofmann, 2009; Dennis *et al.*, 2014; mussels, Jansen *et al.*, 2007; porcelain crabs, Stillman and Tagmount, 2009; and urchins, Osovitz and Hofmann, 2005; see reviews Vernberg, 1962; Sanford and Kelly, 2011); however, most of these studies have focused on heat tolerance. Local differences in cold tolerance remain understudied in marine invertebrate fauna, especially for intertidal organisms (but see Hilbish, 1981; Jansen *et al.*, 2007; Stillman and Tagmount, 2009; Dennis *et al.*, 2014). Here, we expand these investigations to include local differences in the cold tolerance of a broadly distributed intertidal arthropod.

We used the harpacticoid copepod *Tigriopus californicus* as a model to determine whether populations exhibit latitudinal differences in cold tolerance. *Tigriopus californicus* is an ideal study species because individuals have a relatively short generation time (∼20 days) and are easily cultured in laboratory settings (Powlik *et al.*, 1997; Raisuddin *et al.*, 2007). In addition, the species has a broad habitat range, spanning over 30° of latitude from northern Mexico to southern Alaska (Dethier, 1980; Ganz and Burton, 1995), and there is little gene flow between neighbouring populations (Burton *et al.*, 1979; Burton and Feldman, 1981; Burton, 1997; Willett, 2011). As a result, separate populations of *T. californicus* adapt differentially to local environmental conditions, and strong genetic divergence has been observed between isolated rock pools (Burton and Lee, 1994; Ganz and Burton, 1995). Previous work has shown that the heat tolerance of *T. californicus* increases with decreasing latitude (Willett, 2010; Kelly *et al.*, 2011; T. L. Kim, G. T. Wallace and C. J. Neufeld, unpublished data), but no studies have investigated whether a similar pattern exists for this species' resistance to cold temperatures. By examining the cold resistance of a species for which a known latitudinal trend in heat tolerance exists, we can also consider potential physiological trade-offs in the thermal tolerance ranges of populations living in different environmental conditions (Huey and Kingsolver, 1993).

Several ecologically relevant assays exist for evaluating thermal tolerance in ectotherms. In many species, low temperatures induce a state of reversible dormancy several degrees above the lethal low temperature (see review by Hazell and Bale, 2011). This state of narcosis, called chill-coma, is characterized by complete immobility and a large decrease in metabolic function (see review by MacMillan and Sinclair, 2011). Measurements of the temperature which induces chill-coma [critical thermal minimum (CT_min_)] and the time it takes for individuals to recover from this state [chill-coma recovery time (CCR)] are commonly used to make comparisons of cold tolerance across taxa (Huey *et al.*, 1992; David *et al.*, 1998; Ransberry *et al.*, 2011; Sinclair *et al.*, 2012). Several studies have demonstrated that there are latitudinal trends in the CT_min_ (*Drosophila*: Gibert and Huey, 2001; Overgaard *et al.*, 2011a, b) and CCR (*Drosophila*: Gibert *et al.*, 2001; Hoffmann *et al.*, 2001, 2002; Hallas *et al.*, 2002; David *et al.*, 2003; Sisodia and Singh, 2010; Overgaard *et al.*, 2011a, b; isopods: Castañeda *et al.*, 2004, 2005) of terrestrial arthropods; however, no studies have used chill-coma metrics to compare populations of marine arthropods. An additional assay for comparing interpopulational cold tolerance is post-freezing recovery. *Tigriopus californicus* inhabits small upper-shore rock pools in the intertidal zone that are often isolated from the ocean for several days at a time (Burton *et al.*, 1979; Powlik *et al.*, 1997). As a result, these pools can experience extreme variations in temperature on both a daily and a seasonal basis, and populations at northern latitudes must occasionally deal with ice forming in their pools (McAllen and Block, 1997). Monitoring of recovery rates and patterns following exposure to freezing water allows for comparison of cold-recovery abilities. Although none of these metrics have been tested in *T. californicus*, two closely related species, *Tigriopus brevicornis* (McAllen *et al.*, 1999) and *Tigriopus japonicus* (Kasahara and Akiyama, 1976), have been shown to enter chill-comas, and *T. brevicornis* survives freezing by supercooling to avoid the formation of internal ice crystals (McAllen and Block, 1997).

We used CT_min_, CCR and post-freezing recovery as three separate metrics to compare cold resilience in *T. californicus* populations from a wide geographical range. Organisms tend to develop only the level of adaptive response needed to meet an existing ecological challenge; the ability to tolerate an increased level of environmental stress will not develop unless a long-term change in the environment necessitates it (Slobodkin and Rapoport, 1974). Given this and previous studies demonstrating greater heat tolerance in populations of *T. californicus* from warmer environments, we hypothesized that the physiological mechanisms associated with cold resistance may exhibit a cost and would therefore be selected for only in populations that experience very low temperatures on a yearly basis (Pörtner, 2002a; Somero, 2002; Kelly *et al.*, 2013). We predicted, therefore, that populations from colder, higher latitudes would display greater cold tolerance than those from more southern localities and hence would show thermal tolerance traits that closely match their habitat's environmental conditions.

## Materials and methods

### Collection and maintenance of experimental copepods

**Figure 1:**
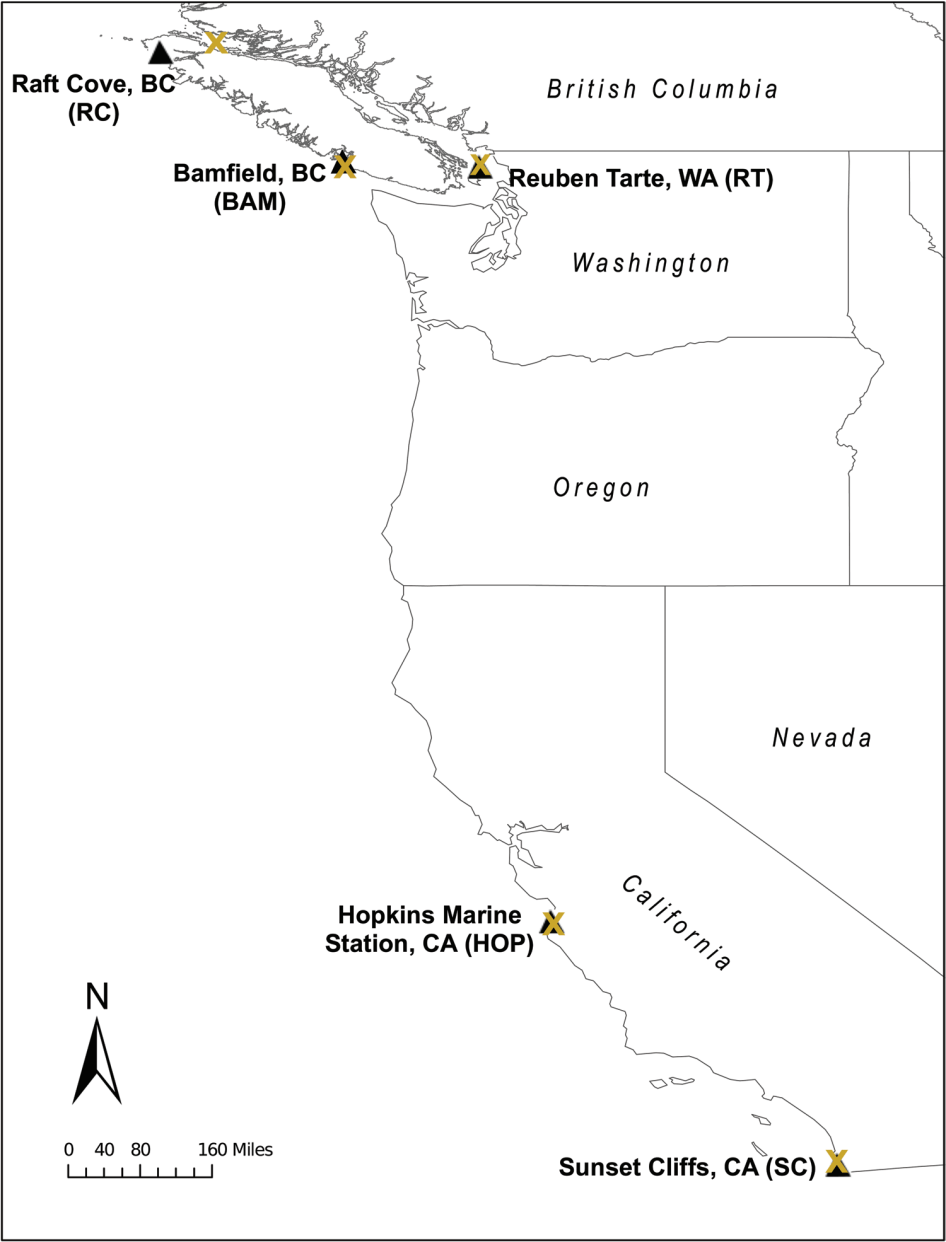
Collection sites of *Tigriopus californicus* along the Pacific coast of North America*.* Triangles represent collection sites, and site abbreviation codes are noted in parentheses. Crosses represent the nearest coastal weather station to each location, from which air-temperature data were obtained for each site (Table 1). Collection sites listed in order of decreasing latitude are as follows: RC, Raft Cove Provincial Park, BC, Canada, 50°58′ N, 128°23′ W; BAM, Bamfield Marine Sciences Centre, Bamfield East, BC, Canada, 48°83′ N, 125°14′ W; RT, Reuben Tarte, San Juan Island, WA, USA, 48°61′ N, 123°10′ W; HOP, Hopkins Marine Station, Pacific Grove, CA, USA, 36°62′ N, 121°90′ W; and SC, Sunset Cliffs, San Diego, CA, USA, 32°43′ N, 117°15′ W. From north to south, weather station locations are as follows: 50°70′ N, 127°49′ W; 48°61′ N, 122°83′ W; 48°50′ N, 125°07′ W; 36°59′ N, 121°85′ W and 32°76′ N, 117°22′ W.

In order to compare populations from a broad environmental range, *T. californicus* specimens were collected from five locations spanning 18°N from southern California to Vancouver Island, BC, Canada (Fig. 1). Air-temperature data from the most proximate coastal weather station to each collection site was used to estimate the thermal conditions of each location (Table 1). While it is unlikely that air temperature provides an accurate representation of upper-shore rock pool temperature, monitoring of the pools that *T. californicus* inhabits in central California indicates that these bodies of water typically are slightly warmer than the air (Egloff, 1966). No sites from southern Washington and Oregon were selected, because previous analyses of heat tolerance in *T. californicus* showed smaller differences between northern populations than between those from southern latitudes (Edmands, 2001; Kelly *et al.*, 2011; T. L. Kim, G. T. Wallace and C. J. Neufeld, unpublished data).

**Table 1:** Climatic data for the collection sites of *Tigriopus californicus* specimens used in this study

Site	Winter daily minimum (°C)	Summer daily maximum (°C)	Number of days ≤0°C
Raft Cove	1.51 ± 0.23	17.28 ± 1.21	56.23
Bamfield	1.94 ± 0.35	18.07 ± 1.51	52.70
Reuben Tarte	1.83 ± 0.44	20.94 ± 1.15	38.50
Hopkins	5.85 ± 0.74	18.91 ± 0.71	1.50
Sunset Cliffs	8.63 ± 0.67	21.72 ± 1.03	0.00

Data were obtained from the coastal weather station closest to each location (Fig. 1). Daily values are reported as mean air temperature values from 1981 to 2010 ± standard deviation for winter (December, January and February) and summer months (June, July and August). The annual mean number of days for which each location experiences freezing conditions is also reported. Temperature data came from the National Climate Data and Information Archive from Environment Canada (http://climate.weatheroffice.gc.ca/climate_normals/index_e.html) for the two Canadian sites and from the National Climatic Data Center (http://www.ncdc.noaa.gov/oa/ncdc.html) for the three American localities (Willett, 2010).

Specimens were collected during late May 2012 from three to five different pools within 100 m of each other at each site and transported to the University of Washington's Friday Harbor Laboratories in Friday Harbor, WA, USA. Five replicate cultures for each collection site were established by placing 25 gravid females into plastic vials containing 100 ml of seawater that had been passed through a 0.45 μm in-line filter. Each culture was started no more than 7 days after the initial specimen collection. All generations were kept in the same vial, and there were no observable differences in the population density of each culture, both between and among collection site replicates. Laboratory cultures were kept in an incubator with a 12 h–12 h light–dark cycle; temperature was maintained at 22°C for the first month of incubation and was decreased to 19°C prior to the start of experimental trials. This was done due to restraints on the availability of laboratory incubator space. The 3°C temperature decrease was not expected to influence the thermal performance of *T. californicus* because this copepod exhibits low plasticity for heat tolerance when exposed to 9°C differences in incubation temperature (Kelly *et al.*, 2011). Copepod cultures were fed TetraMin Tropical Flakes fish food *ad libitum*, and ∼70% (65–70 ml) of the water was changed weekly (Powlik *et al.*, 1997). In our experiments, no cultures were tested until at least 40 days after their establishment. This ensured that a minimum of two generations had grown and developed in the controlled laboratory settings, and all experimental copepods spent their entire lives in their respective culture containers. Thus, the potential confounding effects of environmental plasticity and maternal effects were eliminated, and differences between populations could be attributed to genetic adaptation (Sanford and Kelly, 2011).

### Critical thermal minimum

**Figure 2:**
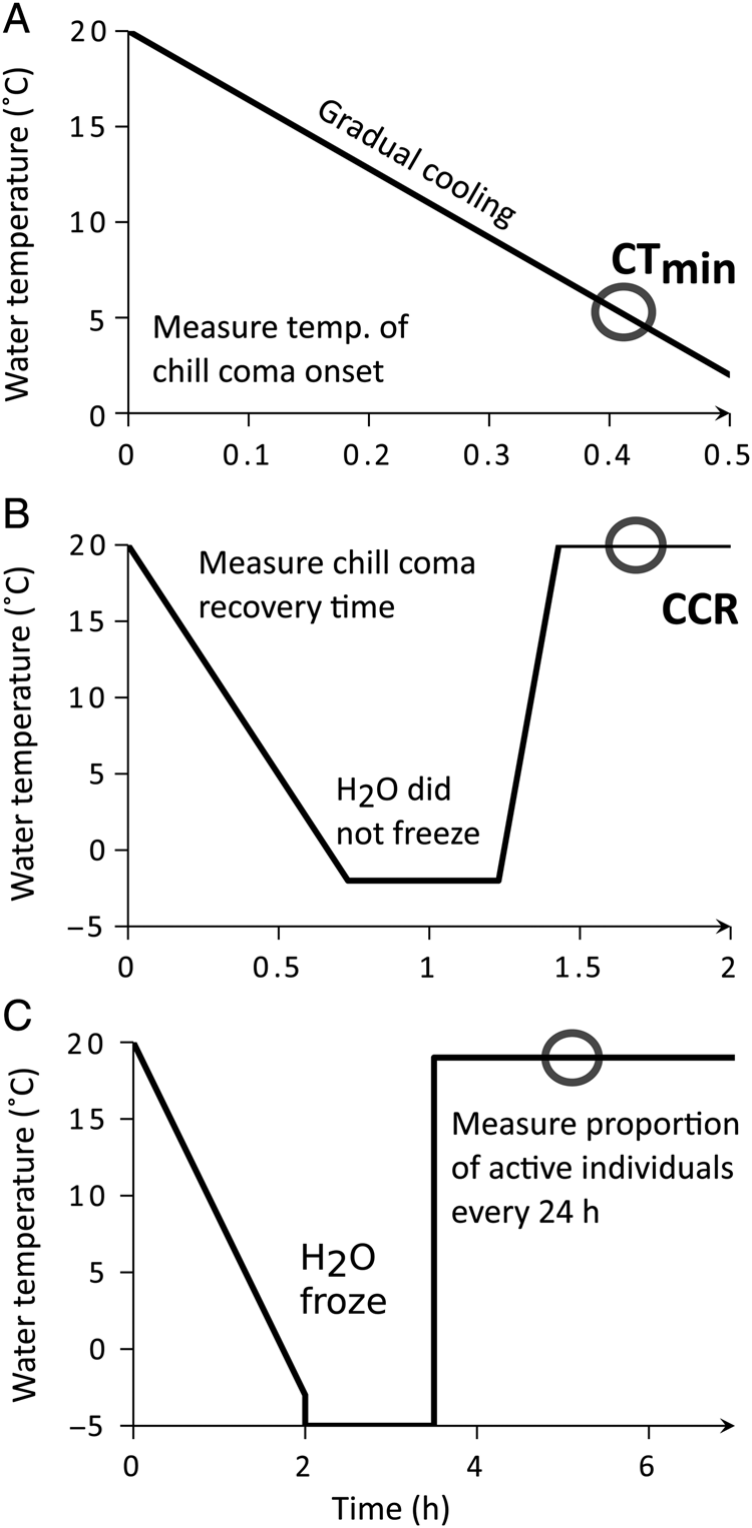
Graphical portrayal of the experimental protocols for scoring each metric of cold tolerance (Overgaard *et al.*, 2011a, b). Lines represent water temperature throughout each experiment, and circles show the measurements made in each assay. (**A**) Critical thermal minima. Copepods were cooled, and the temperature at which 50% of individuals lost mobility was recorded. (**B**) Chill-coma recovery. Copepods were cooled and then held at −2.0°C for 30 min, during which time all individuals entered into a chill-coma. Copepods were then returned to 20°C, and the time needed for 50% of individuals to regain mobility was recorded. (**C**) Post-freezing recovery. Temperature was decreased from 20 to −3°C and then held at −5°C for 90 min, during which time the water froze. Copepods were then returned to 19°C, and the proportion of individuals to recover and demonstrate mobility was recorded at 24 h observation points for 5 days.

In order to measure the critical thermal minima of different *T. californicus* populations, individuals were observed as they cooled down, and the temperature at which they entered into a chill-coma was recorded (Fig. 2A). To remove the potential confounding effects of gender and life stage, only adult males were tested and each copepod was only used once. The adult males were removed from their culture containers using 3 ml transfer pipettes and placed into Petri dishes (30 mm ×10 mm) containing 1.0 ml 30‰ salt water prepared from a mixture of reverse osmosis (RO) water and Instant Ocean Mix. Although the filtered seawater used for each copepod culture came from the same source and was likely to have consistent salinity, Instant Ocean Mix was used as an extra measure to ensure that salinity was controlled exactly in experiments. For this and all other copepod transfers described below, we controlled for salinity by briefly placing individuals on 0.45 μm filter paper to remove excess water before moving them into the experimental dishes. Eight adult males were tested from each of four replicate cultures for every population (i.e. 32 individuals for each collection site). The Petri dishes sat on top of a thermoelectric chiller/heater (Model CP-065; TE Technologies, Traverse City, MI, USA), and the whole apparatus was positioned under a dissecting microscope so that observations could be made throughout each trial. The thermoelectric chiller/heater was then manually programmed to cool the water from room temperature (∼20°C) to 0°C at a rate of −0.5°C/min. Individuals were observed constantly throughout the cooling process, and the temperature at which 50% of individuals entered into a chill-coma (CT_min50_) was measured using a thermocouple. Here, chill-coma was defined as complete immobility, and individuals were considered to be in this state of dormancy after 10 s without any twitching of their legs or antennae.

### Chill-coma recovery

To measure the chill-coma recovery times of different *T. californicus* populations, copepods were put into a chill-coma, and the time it took to recover from this dormant state was measured (Fig. 2B). Again, four replicate cultures from each collection site were used and only adult males were tested. Eight adult males from one replicate were pipetted into a Petri dish (30 mm ×10 mm) containing 2 ml 30‰ seawater. The dish was placed on top of the thermoelectric chiller/heater described above, but Styrofoam walls and a lid were added for insulation. The temperature of the water was decreased from room temperature to −2.0°C at a rate of −0.5°C/min and then held at −2.0°C for 30 min. During this cooling period, the seawater did not freeze and every copepod entered into a chill-coma. The Petri dishes were then immediately returned to room temperature and positioned under a dissecting microscope, where the adult males were observed as the water temperature rose (increased at a rate of 2°C/min). The time at which 50% of individuals had recovered from their chill-coma (CCR_50_) was recorded. Copepods were considered to have recovered when they were able to move their body through the water in response to a mild disturbance (gentle, continuous rotating of the Petri dish). Although some copepods may have been in different physiological levels of narcosis because the chill-coma onset temperature varied (Macdonald *et al.*, 2004), we used this uniform-temperature protocol to compare how populations responded to the same environmental stress.

### Post-freezing recovery

Individuals from each collection site were frozen in seawater and their ability to recover was monitored (Fig. 2C). Twelve adult males were tested from each of four replicate cultures for every collection site (i.e. 48 individuals for each collection site). The copepods were transferred into 0.2 ml microcentrifuge tubes, with four individuals and 0.11 ml 30‰ seawater (again prepared from RO water and Instant Ocean Mix) in each tube. These were cooled down from 20 to −3°C at a rate of −0.2°C/min in a thermocycler (Model T1 Gradient; Biometra, Göttingen, Germany). The adult males were then transferred out of the microcentrifuge tubes and placed into Petri dishes (30 mm ×10 mm) on top of the Styrofoam-insulated thermoelectric chiller/heater. All 12 copepods from each replicate were combined into one dish, and each dish contained 2 ml 30‰ seawater that had been previously chilled to −3°C. The temperature of the water was then decreased to −5°C, and the dishes containing the copepods were left on the cold source for 90 min, during which solid ice crystals formed throughout the solution. Immediately after the freezing period, the copepods were returned to their incubator set at 19°C. The abrupt return to 19°C was not expected to be deleterious because the temperature of the rock pools that *T. californicus* inhabits can increase rapidly with incoming tides and intense sunlight (C. J. Neufeld, unpublished data). For the next 5 days each dish was removed from the incubator every 24 h and the copepods were examined under a dissecting microscope; the proportion of recovered individuals was recorded. Thus, every dish and all copepods were monitored daily throughout the 5 day period. Copepods were considered recovered when they were able to propel their bodies through the water, and each observation point measured the proportion of active vs. inactive individuals in each dish. It was assumed from preliminary trials that individuals remaining immobile after the 120 h recovery period were dead, and the proportion of individuals that survived the freezing was recorded in the final observation. Dead copepods were not removed until the end of the trial period because they could not be distinguished from dormant individuals that could recover.

### Statistical analysis

For all experiments, we analysed interpopulational variation using a one-way ANOVA accompanied by a *post hoc* Tukey–Kramer HSD test. Linear regressions were also used to test the relationship between each metric of cold tolerance and the average winter daily minimum temperature and annual number of freezing days of each collection site (Table 1). Winter minimum air temperature was selected as a primary temperature proxy because the temperatures that induced chill-coma mainly occur during winter months. All analyses were performed in R v. 3.0.1.

## Results

### Chill-coma onset temperature and recovery time

**Figure 3:**
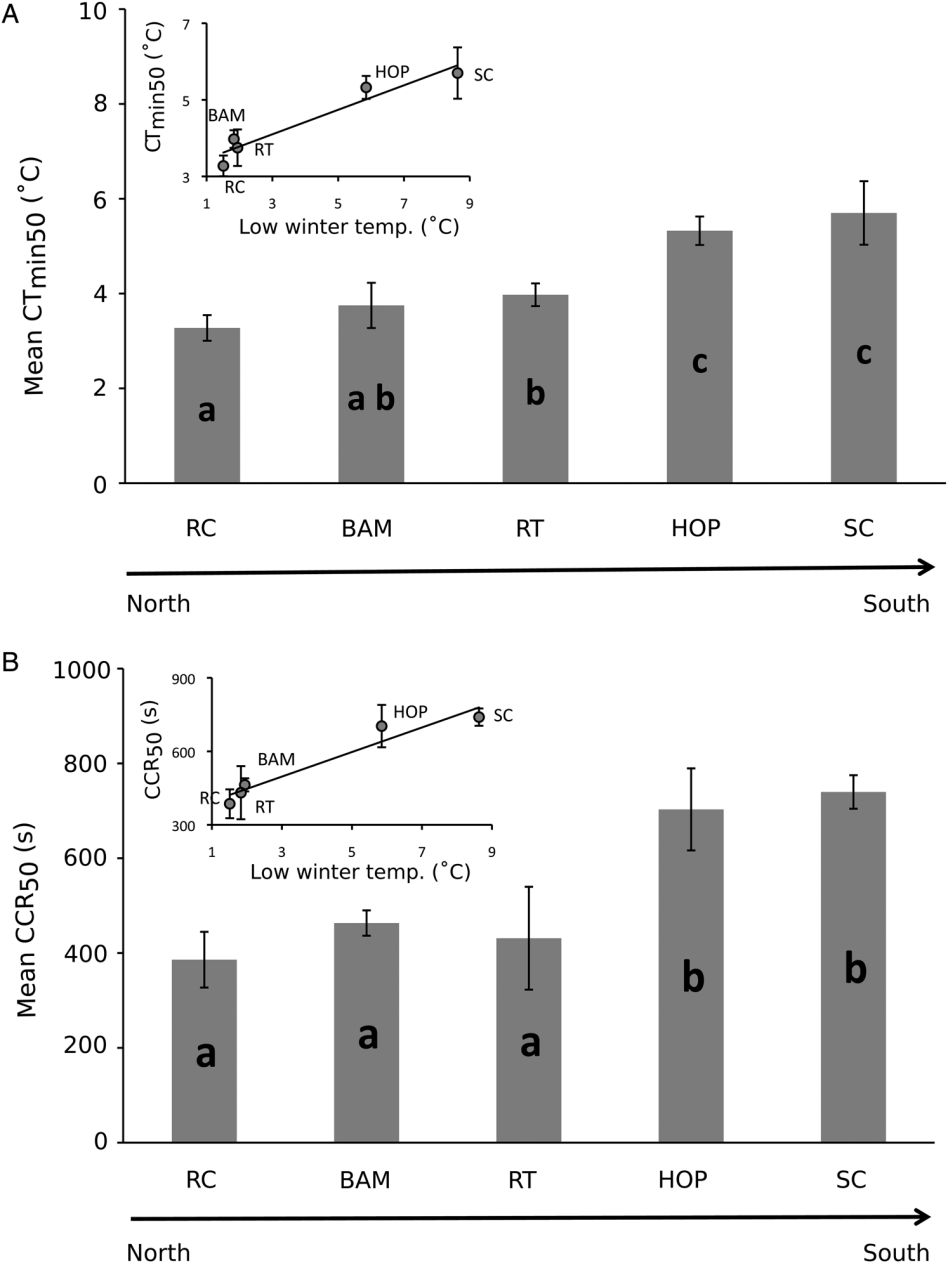
Chill-coma characteristics of *Tigriopus californicus* copepod populations. Populations are listed in order of decreasing latitude and are abbreviated as follows: RC, Raft Cove; BAM, Bamfield; RT, Reuben Tarte; HOP, Hopkins; and SC, Sunset Cliffs. Data are means ± 95% confidence intervals, and letters in bars indicate populations whose means differ significantly from one another (Tukey–Kramer HSD, *P* < 0.05). (**A**) Chill-coma onset temperature for 50% of individuals (CT_min50_). Copepods from northern latitudes entered into a chill-coma at significantly lower temperatures than those from more southern locations. Inset, a regression analysis showed a positive linear relationship between colder natural habitat and ability to remain active in cooling water. (**B**) Chill-coma recovery time for 50% of individuals (CCR_50_). The two southern populations had significantly longer recovery times than those from colder, northern latitudes. Inset, copepods from cold natural habitats regained movement faster than those from warmer regions.

Individuals from northern populations entered into a chill-coma at significantly lower temperatures than those from warmer southern populations (*F*_4,15_ = 61.738, *P* < 0.001; Fig. 3A). Furthermore, CT_min50_ was negatively correlated with the average winter minimum temperature (*P* = 0.004, slope = 0.320, *r*^2^ = 0.927; Fig. 3A) and mean annual number of freezing days (*P* < 0.001, slope = −0.038, *r*^2^ = 0.972) of each collection site. Likewise, chill-coma recovery time (CCR_50_) increased significantly with increasing average minimum winter temperature (*P* = 0.004, slope = 50.126, *r*^2^ = 0.932; Fig. 3B) and annual number of freezing days (*P* = 0.004, slope = −5.804, *r*^2^ = 0.934) of each collection site. The two southern-most populations had significantly longer chill-coma recovery times than those from northern populations (*F*_4,15_ = 55.211, *P* < 0.001; Fig. 3B).

### Post-freezing recovery

**Figure 4:**
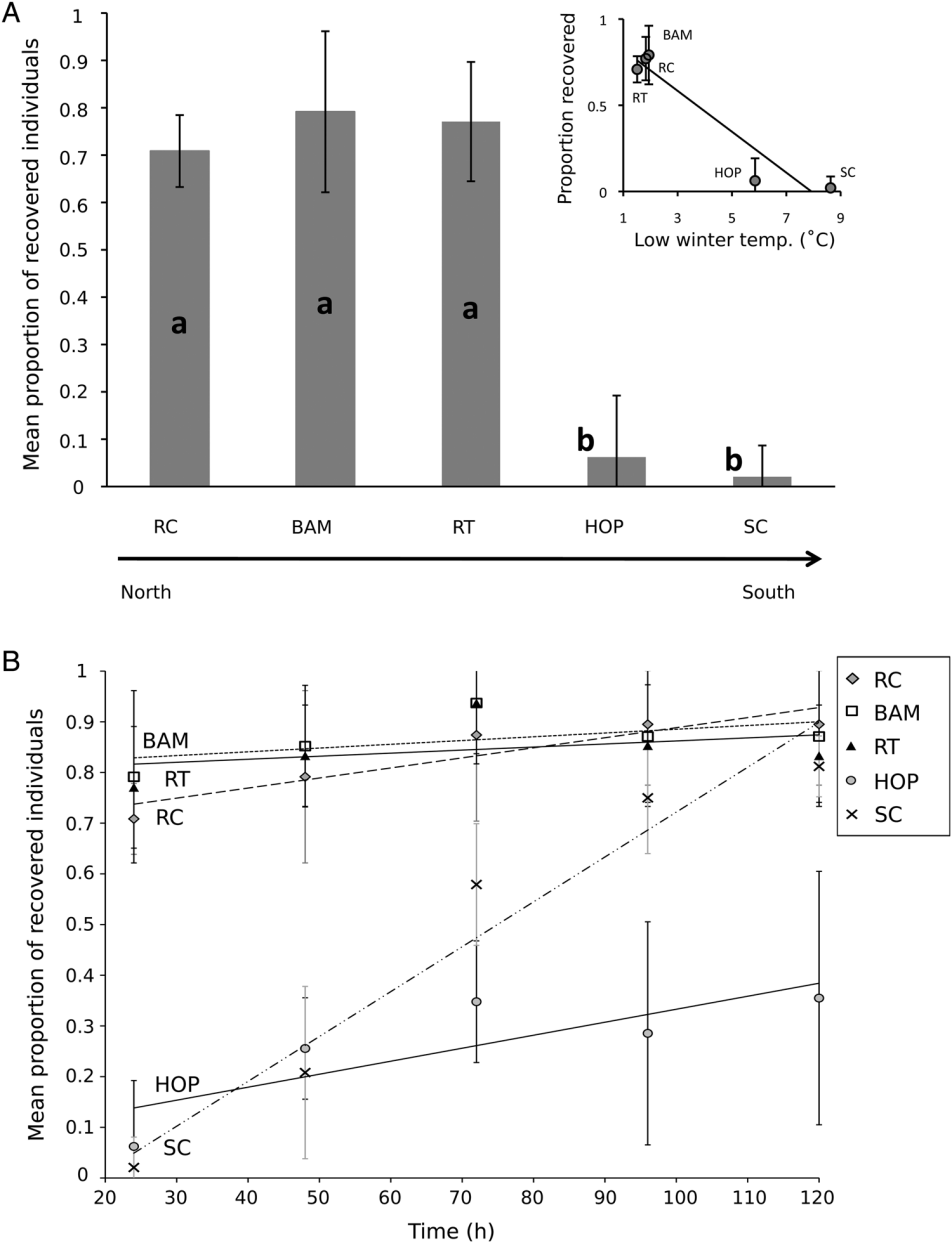
Post-freezing recovery of *Tigriopus californicus* copepod populations. Populations are listed as in Fig. 3. Data are means ± 95% confidence intervals, and letters in bars indicate populations whose means significantly differ from one another (Tukey–Kramer HSD, *P* < 0.05). (**A**) Proportion of individuals that recovered 24 h after being in frozen water. The three northern populations had significantly larger proportions of recovered individuals than the two southern populations. Inset, copepods from colder natural habitats recover more quickly from ice-forming conditions than those from warmer regions. (**B**) Five day post-freezing recovery patterns of each population. There were significant differences between collection sites, with Sunset Cliffs displaying a much steeper recovery trajectory than the other four populations.

Twenty-four hours after exposure to frozen water, >70% of the copepods from the each of three northern populations had regained mobility, whereas <10% of each Californian population had recovered (*F*_4,15_ = 131.446, *P* < 0.001; Fig. 4A). In addition, the mean proportion of recovered individuals after 24 h was negatively correlated with the average winter minimum temperature (*P* = 0.006, slope = −0.118, *r*^2^ = 0.909; Fig. 4A) and annual number of freezing days (*P* = 0.005, slope = 0.013794, *r*^2^ = 0.919) of each collection site. Post-freezing recovery trajectories (the slope of the proportion-recovered line for each population over 5 days) also differed across latitude (*F*_4,15_ = 32.435, *P* < 0.001; Fig. 4B), but recovery trajectories were not significantly correlated with the average climatic conditions at each collection site. Most copepods from the three northern populations (Reuben Tarte, Bamfield and Raft Cove) recovered within the first 24 h after freezing. The Hopkins copepods from Central California had low recovery rates and high mortality, with only 35% surviving the frozen water. The southernmost population, Sunset Cliffs, had a similarly low initial recovery rate, but exhibited a steep recovery trajectory such that the final proportion of recovered individuals was similar to those from colder climates.

## Discussion

Populations of *T. californicus* that experience colder temperatures in their natural habitats had greater cold tolerance than those from warmer climates. The copepods exhibited significant positive latitudinal clines in both CT_min_ and CCR (Fig. 3). In addition, in comparison to a >70% recovery rate in northern copepods, a significantly lower proportion of the southern individuals recovered within 24 h after having been in frozen water, indicating a reduced ability to respond adaptively to cold (Fig. 4A). In an environmental context, each metric was significantly related to the average low winter temperature and annual number of freezing days of the collection site of each population, suggesting directional selection for the traits associated with cold tolerance (Castañeda *et al.*, 2005). In all three metrics, *T. californicus* entered into a state of narcosis. Dormant animals are more susceptible to pathogens and predation, and narcosis interrupts activities such as feeding and reproduction (Powell and Bale, 2006); therefore, remaining active in cold water for as long as possible is likely to be adaptive, and the northern populations of *T. californicus* were more functional at lower temperatures than copepods from lower latitudes. Our results add to a growing body of data showing that populations within a single species can display strong local adaptation to spatially varying climatic conditions. Importantly, all three metrics showed a consistent latitudinal trend, indicating that any one could be used equivalently in future studies investigating latitudinal variation in cold tolerance (similar to Overgaard *et al.*, 2011a; Ransberry *et al.*, 2011).

Five day post-freezing recovery patterns also suggest greater cold tolerance in *T. californicus* from colder environments, but the results are less consistent than those from the CCR, CT_min_ and the 24 h post-freezing measurements (Fig. 4B). Most of the copepods from the three northern collection sites (Reuben Tarte, Bamfield and Raft Cove) recovered within the first 24 h after freezing and had few fatalities. Individuals from these collection sites that did not recover within 24 h were unlikely to recover at all. In contrast, the Sunset Cliffs population had a low initial recovery rate, but by the end of the 5 day observation period the final proportion of recovered individuals was comparable to those from colder climates. The Hopkins copepods from Central California had a high mortality rate, with <40% of individuals surviving the stress. The fact that a small proportion of the Hopkins copepods survived indicates that the traits necessary for either avoiding or tolerating internal ice-crystal formation were present in the population but were not found in most of these individuals.

Given that the Sunset Cliffs copepods had much longer post-freezing recovery periods than the higher latitude populations, future research could investigate whether *T. californicus* from this collection site (and other low latitudes) use the same mechanisms for entering into/recovering from freezing-induced dormancy as northern copepods that regain motion within 24 h after the cold stress. For example, a lower latitude population of *T. californicus* demonstrated greater heat shock protein expression in response to heat stress than a higher latitude population (*hsp70*; Schoville *et al.*, 2012). As the heat shock response is involved in a wide variety of environmental stresses, including cold stress (Feder and Hofmann, 1999), southern populations might rely on heat shock proteins as molecular chaperones to prevent cellular damage from both heat and cold stress. While northern populations may also exhibit the heat shock protein response, the different recovery patterns could be explained by different physiological capabilities among populations.

*Tigriopus brevicornis*, a closely related species with a broad geographical range in Europe, supercools to avoid internal freezing (McAllen and Block, 1997). In supercooling, *T. brevicornis* lowers its internal freezing point below that of water, probably by osmoconforming to increasingly saline water as ice crystals form in their environment (Damgaard and Davenport, 1994); therefore, *T. brevicornis* might rest in small areas of highly saline liquid water between ice crystals. *Tigriopus californicus* also increases its internal osmolyte concentration when acclimatized to high salinities (Burton and Feldman, 1982). While all *T. californicus* populations osmoconform as their rock pools evaporate or collect water, copepods that experience freezing in their natural environment may be able to upregulate their internal solute concentration more rapidly in cold conditions to avoid freezing as ice crystals form in surrounding water. Cold-specific adaptations may enable a faster recovery and are more likely to be selected for in northern populations that experience cold conditions on a regular basis. Thus, a possible explanation for the observed interpopulational differences in post-freezing recovery could be a greater reliance on heat shock proteins in southern populations vs. more dependence on cold-specific adaptations (such as supercooling) in northern populations.

The exact mechanisms of chill-coma onset and recovery are still unknown (MacMillan and Sinclair, 2011), and neither has been studied in a marine organism. In addition, the mechanisms of post-freezing recovery of *T. californicus* are poorly understood. For example, it is notable that some of the Sunset Cliffs copepods were able to survive almost 120 h in a dormant state after freezing, but it is not known whether this freezing-induced dormancy has the same underlying mechanisms as chill-coma. Although the lethal low temperature for *T. californicus* is unknown, *T. brevicornus* tolerates temperatures down to −16.9°C (Damgaard and Davenport, 1994), indicating that this genus can physiologically tolerate temperatures well below freezing. Thus far, most studies investigating arthropod cold physiology have used *Drosophila* spp. as a model system (Hoffmann, 2010). However, while *Drosophila* makes an excellent study species for cold physiology in terrestrial arthropods, there is no comparable model for studying cold physiology in intertidal arthropods. While several intertidal arthropods are known to tolerate freezing conditions (e.g. Waller *et al.*, 2006; Ronges *et al.*, 2012), *T. californicus* has been well studied and has the properties of an ideal *in vivo* model system. Like *Drosophila*, *T. californicus* is small, has a short generation time, is easily cultured in laboratory conditions and demonstrates latitudinal clines in CCR and CT_min_ (Hoffmann, 2010). *Tigriopus californicus* has been used as a model system for studying a broad range of topics, including population genetics (e.g. Pritchard and Edmands, 2013), environmental toxicity (e.g. Misitano and Schiewe, 1990) and evolutionary neuroscience (e.g. Andrew *et al.*, 2012). We propose that *T. californicus* could also be a useful model for investigating mechanisms of arthropod cold tolerance. Given that intertidal organisms experience different types, amounts and rates of environmental changes from terrestrial taxa (Clarke, 2009), there is a need for such a model organism.

While the breadth of thermal tolerance tends to increase with latitude in terrestrial taxa (Addo-Bediako *et al.*, 2000), recent analyses suggests that the breadth of thermal tolerance of marine organisms is fairly consistent across latitude, despite changes to both upper and lower thermal limits (Sunday *et al.*, 2010). The cold tolerance of *T. californicus* increased significantly with latitude, while previous studies have shown that the heat tolerance of this species decreases significantly with latitude (Willett, 2010; Kelly *et al.*, 2011; T. L. Kim, G. T. Wallace and C. J. Neufeld, unpublished data). Our results, in conjunction with those of Willett (2010) and Kelly *et al.* (2011), suggest that instead of expanding or shrinking across latitudes, the thermal performance windows of *T. californicus* appear to shift in adjustment to local conditions across environmental gradients, hence supporting Sunday *et al.* (2010) in showing similar thermal tolerance breadths across latitude in marine animals. Thus, there appear to be physiological trade-offs in the thermal tolerance of *T. californicus* from different latitudes, because cold-adapted populations have lower heat tolerance, while populations from warmer climates are less cold resistant. Willett (2010) observed performance trade-offs between *T. californicus* populations at two ambient temperatures. While there does not appear to be a thermal cost for increased lethal high temperature tolerance in *T. californicus* (Kelly *et al.*, 2013), at least in terms of fecundity, body size and starvation resistance, future studies measuring both upper and lower thermal critical values of different populations could investigate whether there are performance trade-offs between traits necessary for heat vs. cold tolerance in this species (Hoffmann *et al.*, 2002).

Given that ramping rates and laboratory conditions can affect critical values and chill-coma length, the experimental values we recorded may not reflect the true performance of *T. californicus* in their natural environment (Terblanche *et al.*, 2007). Nonetheless, a comparison of chill-coma onset values with collection site annual low winter temperature shows that northern copepods enter into chill-coma at slightly warmer temperatures than the mean low temperature of their environment, while southern individuals enter chill-coma at temperatures slightly below the winter minimum temperature of their habitat. This suggests that the northern populations live closer to their limits of cold tolerance, while southern populations may live closer to their guardrails of heat tolerance (Hutchins, 1947; Kelly *et al.*, 2011). *Tigriopus californicus* may therefore be sensitive to changes in the minimum and maximum temperatures of its habitat: higher latitude populations may be less able to tolerate decreasing minimum temperatures, while southern copepods may be more sensitive to increasing maximum temperatures. Interestingly, southern *T. californicus* exhibit high levels of genetic divergence over fine geographical scales, while northern populations appear more closely related to each other (Edmands, 2001). Our uniform protocols showed differences between the thermal performance of populations along a broad latitudinal scale, but the exact nature of the relationship between cold tolerance and latitude could be clarified through more intensive spatial sampling of *T. californicus* populations, both locally and on a broad scale (Sanford and Kelly, 2011).

Strong local adaptation patterns may also have implications for understanding biological responses to anthropogenic climate change. The thermal performance of *T. californicus* is sensitive to environmental differences along a spatial gradient, indicating that it will probably also be sensitive to environmental changes over time. In addition to predicting an overall increase in global temperature, climate models forecast widespread changes to both the maximum and the minimum temperatures of several regions (Easterling *et al.*, 2000). In some cases, this means that organisms will be exposed to lower minimum temperatures on a seasonal basis (Petoukhov and Semenov, 2010). Isolated populations of *T. californicus* have different low-temperature tolerance limits from the species as a whole, meaning that separate populations may respond in a different manner to changing winter minimum temperatures as global climate change progresses. As pointed out by Kuo and Sanford (2009) and Kelly *et al.* (2011), bioclimate envelope models that treat the environmental tolerance of species as fixed throughout their entire habitat range may incorrectly estimate extinction potentials. Future work to identify more precise patterns of macrophysiology and to understand the underlying physiological mechanisms of comparative thermal tolerance (Gaston *et al.*, 2009; Sinclair *et al.*, 2012) will be useful for understanding how broadly distributed species like *T. californicus* will respond to climate change.

## Funding

This work was supported by an National Science Foundation REU-Blinks-BEACON fellowship at The University of Washington's Friday Harbor Laboratories to G.T.W. and a Friday Harbor Laboratories Postdoctoral Fellowship to C.J.N.
